# Improvement of mahogany leaf extract dye fixation on cotton-modal blend

**DOI:** 10.1016/j.heliyon.2023.e20786

**Published:** 2023-10-07

**Authors:** Md. Abdul Hannan, Md. Faridul Islam, Mohammad Bellal Hoque

**Affiliations:** a Department of Textile Engineering, Dhaka University of Engineering and Technology, Dhaka, Bangladesh; b Department of Textile Engineering, World University of Bangladesh, Dhaka, Bangladesh

**Keywords:** Natural dye, Mahogany leaf, Mordantless, Eco-friendly, Color fastness

## Abstract

The manufacture of regenerated cellulose-based fibers for better mechanical and comfort qualities was stimulated by the rising demand for cotton and the low production rate needed to meet global demands. Modal-cotton blend provides better tensile and moisture management properties. The present work has been designed to sketch out the scope of increased dye fixation or dye uptake opportunity onto the blends. Cotton-modal blend was dyed with mahogany leaf extract dyes avoiding mordant. The higher wash fastness rating 4/5, 5 along with the FTIR characteristic bands around 1190-1210 cm^−1^ created attention for the confirmation of dye-fibre bonding. But as modal is a regenerated cellulosic fibre, there was a suspect of uneven fixation because of dual way dye penetration options inside the fibre: direct bonding with cotton cellulose and dye penetration into swollen modal fibre through segmental mobility theory. Fortunately the uniformity of shade was affirmed by the determination of evenness through random CMC DE and K/S values at distinguished parts of the same sample. Mordantless mahogany dye fixation on cotton-modal blend was found even at the elevated dyeing temperature of 130 °C. The detailed CIE Lab data explored the close symmetry and uniformity of the dyeing outcomes of the blend.

## 1 Introduction

The increasing demand of cotton and low production rate to fulfill the world requirements boosted the production of regenerated cellulose-based fibers for higher mechanical and comfort properties. Modal-cotton blend provides better tensile properties and higher moisture management properties [[1], [2], [3]]. Regarding the coloration of the blends, double stage synthetic dyeing is applied. But, due to the growing worldwide environmental concern and human safety, the idea gets flipped back to natural dyeing process, a sustainable approach for textile coloration [[2], [3], [4], [5], [6]]. Again, mordants are used conventionally for the natural dyeing on cotton and the cellulosics. As mordanting agents, different metal salts are used, which are themselves more pernicious to environment than many other synthetic dyes. Henceforth, significant number of attempts have been made to search and applying natural mordants while dyeing with natural colorants [7]. But, few research works have been done on mordantless natural dyeing on cotton where color fastness and dyeing uniformity were not adjusted accurately [8]. But it is important to dye cotton and blends without mordant keeping optimum durability and fastness properties. In this research natural dyes sourced from mahogany (Swietenia mahagoni) leaf extracts will be applied on cotton-modal blend as they consist some useful functional groups that can be attached to the cellulosic blends [9]. Mordant-free dyeing faces problems of low depth of shade due to insufficient bonding of dyes with fibres. To increase the dye fixation several attempts were taken previously. Ultrasonic energy application for increasing synthetic dye fixation was previously appreciated [10]. For increment of natural dye fixation, pressurized hot water extraction (PHWE) method was utilized [7]. Natural dyeing at high temperature has impact on dye fixation [11]. Modal fibre is cellulosic but manmade by preparation, so dye fixation at high temperature will also be assessed by free volume ideology [12]. Optimization of dyeing time and dye concentration for increased dye fixation were also presented in the previous works [13,14]. But very few works could be found on the effect of the mentioned parameters on natural dye uptake on blend fabrics. Natural dyes have great potentiality over synthetic dyes. The importance of natural dyes is increasing day by day due to their eco-friendliness [15]. Natural dyes could be utilized as an alternative to synthetic dyes in fire proof garments, food garments, kids’ apparel, medical uniforms, sports costumes etc. for safety [16]. Natural dyes have also been applied both on synthetic (e.g. polyester etc.) and natural fiber like cotton, jute etc. [[17], [18], [19]].

Moreover, some synthetic dyes are carcinogen and mutagen [20]. Thus, due to the growing worldwide environmental concern and human safety, the idea gets back to natural dyeing process, a sustainable approach for textile dyeing. Recently natural processes during wet process are getting under consideration for the eco-friendliness of the globe, and this way the environmental issues are thought to be minimized. In addition, it provides some functional value to textile materials in terms of finishing such as, antimicrobial, UV blocking, deodorizing [16].

Natural dyeing is not the same as conventional synthetic dyeing, and the performance of organically colored fabric is rather low in terms of color strength and color fastness [21]. Consequently, researchers are continuously struggling to achieve better performance by applying different treatments on textile materials such as mordanting to grow affinity between natural colorants and textile materials. Mordanting can be conducted at several phases of the dying process; for example, pre-mordanting, meta-mordanting, and post-mordanting are carried out before, during, and after dyeing, depending on the performance and process variables [22].

Moreover, Samanta [16] and Agarwal [23] mentioned some promising outcomes avoiding mordants on silk, wool, polyester and polyamide fibers. However, the application of natural dyeing on cellulosic fibers, basically cotton, without mordant is scarcely found. On the other hand, cotton is the most widely used apparel fiber all over the world for its excellent properties; for instance, comfortability, flexibility, dyeability, and versatile applications [24]. But, cotton has relatively poor affinity to natural colorants [25]. Hence, the consideration of natural mordants aging reveals in order to dye cotton with natural colorants.

Mohammad and the authors [8] of the paper realize that natural dyeing of cotton has not been able to fully overcome its limitations. Mamun et al. [26] worked on mordant-free natural dyeing on nylon with mahagony seed-pod.

There are a tremendous number of mahogany plants in Bangladesh which develop a bounty of leaves each year. These leaves have no industrial uses in the growing zones and, subsequently, tons of tons of leaves are essentially wasted. On the other hand, the scientific use of these leaves for textile coloring purposes may be an efficient way to diminish the problem of such waste disposal and, at the same time, financial utilization in textile industries. The mahogany leaf family of woody trees (Meliaceae) is the complete source of triterpenoids, scopoletin, melianone, cyclomehogenol, swietenin, stigmasterol glucose, limonoids etc. [[27], [28], [29], [30]]. The chemical components of mahogany leaf extract are mostly triterpenoids of limonoid class [9], where most triterpenoids are alcohol and can combine with sugars to form glycoside [31]. The repeating unit of cotton is cellubiose, where two ẞ-glucose units are linked with 1–4 glucosidic linkage.

Till dates cotton dyeing with natural sources are done with mordant commonly but it is important to dye cotton without mordant keeping optimum durability and fastness properties. The aim of this research study was to develop an energy saving dyeing method of cotton-modal blended knit fabric with available natural dyes and compare with the conventional one. Effect of elevated temperature, dye concentration and dyeing time on dye uptake of mordant-free mahogany leaf extract dyeing on cotton-modal blend fabric were also investigated in this research.

## 2 Experimental

.

### 2.1 Materials

**Fig. 1:**
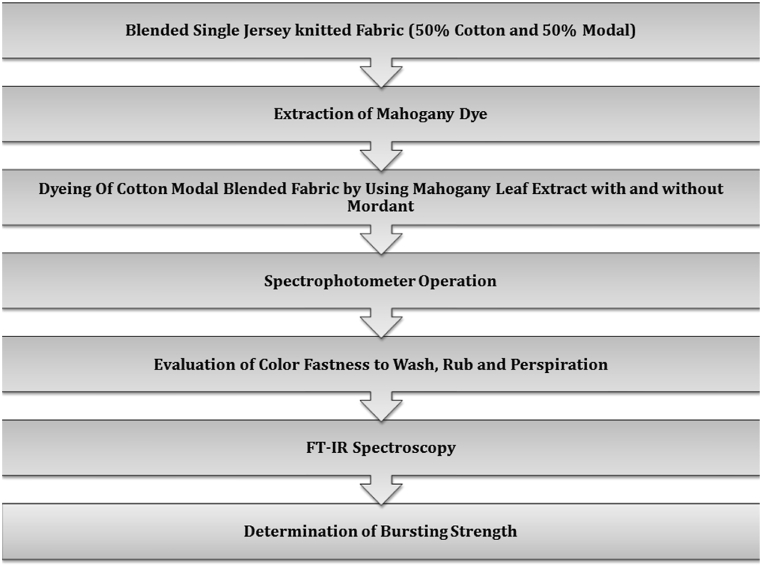
Process flow chart.

50 % cotton and 50 % modal blended single jersey knitted fabric (scoured and bleached) was used for this research work. The GSM of cotton knit was 160. Most important ingredient: natural dye, mahogany leaf/leaves were collected from mahogany trees in nearby DUET area. Dhaka, Bangladesh. Fig. 1 is showing the process flow chart of this work.

### 2.2 Preparation/extraction of mahogany dye

Mahogany leaves were washed very well and blended with water help of blender machine. After blending, the leaf juice was extracted by filtering into liquid form.

### 2.3 Blend fabric and dyeing

The mentioned blend (50 Cotton 50 Modal) knit fabrics were immersed into a dye bath with mahogany leaf extract dye solution. The dyeing temperature was set respectively at 90 °C, 100 °C 110 °C, 120 °C &130 °C for 60 min.

After dyeing, three times hot washes followed by two cold washes were conducted, and then dried.

### 2.4 Chemicals

Copper Sulphate (CuSO_4_) was used as mordant and detergent was used for washing purpose.

### 2.5 Dyeing of cotton modal blended fabric by using mahogany leaf extract (with mordant)

The dyeing procedure was carried out in a standardized atmosphere using natural dyes in an aqueous solution. The weight of the single jersey knit fabric was 10 gm (05 samples was taken) and the coloring process was performed in closed bath process. The sample cotton knit fabric were colored by mahogany dye liquor (90 ml) where CuSO_4_ (1 g/L) was used as mordant and without using any other chemicals or auxiliaries at 90 °C, 100 °C, 110 °C, 120 °C and 130 °C for 60 min or an hour. Here, material-liquor ratio was 1:10 (closed bath). After dyeing, hot wash was performed thrice. Then two times cold wash was done by normal water as if uniform shades perform. Finally, samples were dried by woven dryer machine to eliminate water.

### 2.6 Dyeing of cotton modal blended fabric without using mahogany leaf extract (without mordant)

The sample cotton knit fabric was colored with mahogany dye liquor (100 ml) without the use of any additional chemicals or auxiliaries and also mordant at 90 °C, 100 °C, 110 °C, 120 °C and 130 °C for 60 min or an hour. The material to liquor ratio in this closed bath was 1:10. Hot washes were done after dyeing for three times. Then cold wash was performed twice by using regular water to get uniform shade level. Lastly the samples were finally dried using woven dryer machine to remove the water.

### 2.7 Spectrophotometer Operation

Instrumental color measurement or estimation of the conditioned colored samples were carried out using a Data color 650 spectrophotometer with 10°LAV (large area view) observer using different standard CIE illuminants: D65, TL83 and A. The D65 illuminant represents natural daylight where color temperature is 6500 K. Illuminant TL83 represents tri-band fluorescent (3000 K) and an illuminant represents the incandescent light or tungsten halogen where color temperature is 2856 K. There were two softwares such as-data color tool (DCI- Tool) and data color match (DCI- Match) used to evaluate the measurement of different shades. Reflectance (%) of the colorant samples were also measured by utilizing Data color 650 spectrophotometer in terms of color strength (k/s value).

### 2.8 Evaluation of color fastness to wash, Rub and perspiration

The dyed samples were washed by washing & dry-cleaning color and then the color fastness to wash was tested by a fastness tester; Model-415/8; Brand-James H. Heal; Origin-UK according to ISO 105C10:2006 method. The sizes of samples were 10 × 4 cm. The dyed samples were rubbed by Crock Master; Model-670 hand driven crock master; Brand- James H. Heal; Origin-UK according to EN ISO 105X12:2001 standard under both condition (dry and wet). The sizes of the samples were 10 × 2 cm, arm was weighted to provide a constant 9 N load on the samples at all times and mechanical counter was kept track of completing 10 cycles. Perspiration test (Perspire Meter Phenolic Yellowing Tester & Incubator; Model: HX 30; Brand: James H. Heal; Origin: UK) was performed according to ISO 105-E04. The dimensions of samples for perspiration test were 10 × 4 cm.

### 2.9 FT-IR spectroscopy

Fourier transform infrared spectroscopy (FTIR) provides information related to the presence of specific functional groups. Parameters were utilized in estimations were: resolution 4 cm^−1^, spectral range: 4000-600 cm^−1^ on SHIMADZU-FTIR spectrometer. Omic TM software computer program were performed together and processed the IR spectra. FTIR spectra were recorded specifically from the samples/textile material or fabric. Samples were set on the FTIR spectrums and then were scanned. And the spectra's were obtained in this work collection of FTIR spectra contained presently through and through many spectrums of several samples.

### 2.10 Bursting strength

In this research, ASTM D 3787 ball bursting method was used to calculate bursting strength of the samples where test speed was 300 mm/min, preload 5 N and diameter 45 m and also the required size of the test specimens was (10 cmX10 cm). This methodology evaluates the durability of knit textiles to determine their resistance to burst under multidirectional force. By using a constant-rate-of-traverse tensile tester and a steel ball to tear the fabric, ASTM D3787 is used to calculate the amount of force needed to cause a textile to rupture. A specimen is clamped firmly and tension-free between grooved, circular plates of the ball burst attachment using the pulling (movable) jaw of a constant-rate-of-transverse (CRT) testing equipment. The machine's pendulum-actuating (stationary) clamp presses down on the specimen until it ruptures with the aid of a polished, hardened steel ball.

## 3 Results and discussions

### 3.1 The color strength (K/S) value analysis

**Fig. 2:**
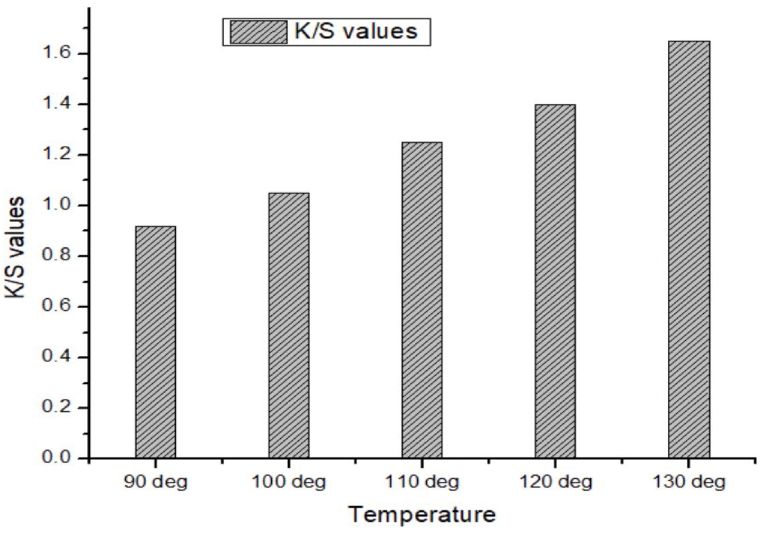
Color strength (K/S) value of mahogany leaf extract dyed sample without using mordant.

**Fig. 3:**
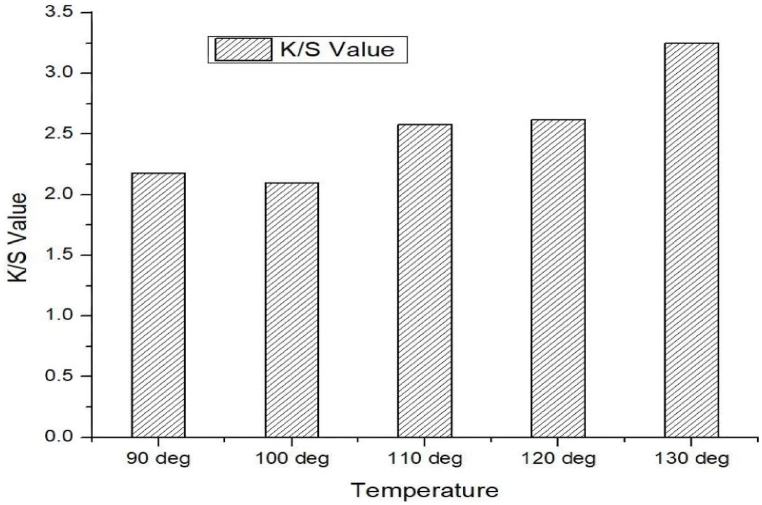
Color strength (K/S) value of mahogany leaf extract dyed sample by using mordant.

From Fig. 2, Fig. 3 it is seen that the K/S value of mahogany leave extract dyed cotton modal blend at temperature 90 °C, 100 °C, 110 °C, 120 °C & 130 °C with mordant were found to be 2.18, 2.10, 2.58, 2.62 & 3.25 respectively. In the same way, the dyed blends without mordant revealed the K/S value of 0.92, 1.05, 1.25, 1.40 & 1.65 respectively.

It is very clear that the mordant treated natural dyed blends received higher values of K/S than mordant less dyed samples. Still it is inspiring that the mordant free mahogany dyed fabric exhibited the value of 1.65 at 130 °C temperature which is close to the values of mordant treated fabrics. It was observed that the depth of shade (K/S value) of the mordantless natural dyed samples increased gradually with the raise of temperature from 90 °C to 130 °C. Mordanted natural dyed samples showed some deviation in the increase of K/S value during the raise of temperature, but fortunately no deviation was experienced for the increase of K/S values during the raise of temperature of the mordantless samples. The proportional linearity in the development of depth of shades for the mordantless samples needs to be understood regarding constitutional components of cotton modal blends. It is known that modal is a semisynthetic fibre which has cellulose as constitutional monomer, and at the same time the same fibre is commonly manufactured following the synthetic fibre production manufacturing pathway. The increment of K/S value in cotton part might have resemblance with the same in modal part. Hopefully the dye uptake behavior and thermodynamics of dyeing of cotton modal blends maintain the same dyeing behavior during the mordant less natural dyeing program.

### 3.2 Evaluation of CIE L* a* b* value

**Fig. 4:**
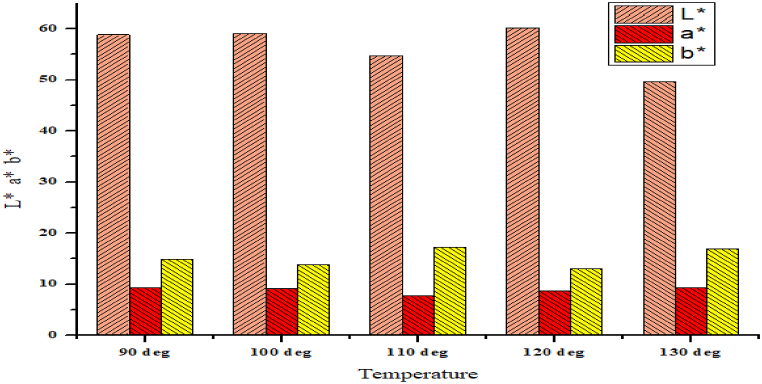
CIE L* a* b* Value of mahogany leaf extract dyed sample by using mordant.

**Fig. 5:**
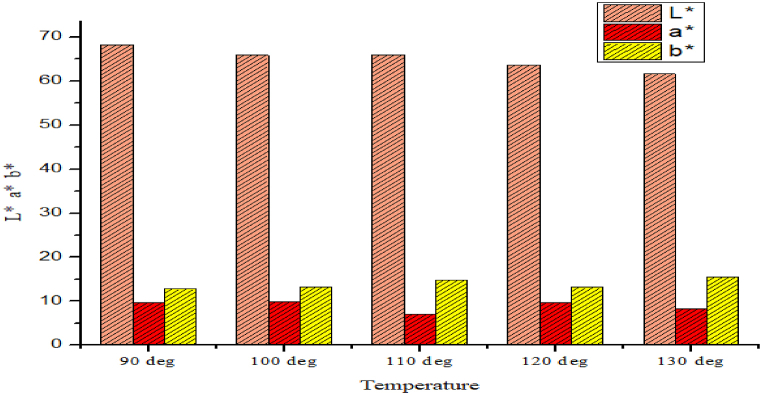
CIE L* a* b* Value of mahogany leaf extract dyed sample without using mordant.

From Table 1, Table 2 and Fig. 4, Fig. 5, The CIE L* a* b* values are depicted/showed to have a look on the colors of the shade. It can be exhibited from the table that the yellow blue scale value increases as the temperature increases, where there is no mordant. The value was initially 12.79 and gradually it increase to 50.50 as per raising of the temperature 90 °C–130 °C. The reason behind the increase of the b* value, it could be that basically the mahogany leaf extract has the color value of pale yellow and sometimes yellow reference something. Because of the raising temperature. Hopefully, more yellow color components are getting attached with the fibre. So, reasonably the b* value is getting higher by the raising of temperature. On the other hand, the red green scale it gets little bit lower during the raising of temperature and of course not that much deviation. So, as the mahogany leaf extract has little effect on red color so there is some deviation but not that much.

**Table 1:** Determination of CIE L* a* b* Value of mahogany leaf extract dyed sample by using mordant.

	Temperature	CIE L* a* b* Values		
		L*	a*	b*
Using Mordant	90 °C	58.86	9.35	14.93
	100 °C	59.05	9.10	13.83
	110 °C	54.73	7.85	17.21
	120 °C	60.17	8.76	12.98
	130 °C	49.63	9.35	16.90

**Table 2:** Determination of CIE L* a* b* Value of mahogany leaf extract dyed sample without using mordant.

	Temperature	CIE L* a* b* Values		
		L*	a*	b*
Without Using Mordant	90 °C	68.26	9.79	12.79
	100 °C	65.93	9.94	13.18
	110 °C	65.94	7.02	14.84
	120 °C	63.69	9.79	13.13
	130 °C	61.68	8.26	15.50

Again, it can be experienced for the mordant treated samples that the yellow blue scales has some rapid increment and then less increment of the yellow scale. The reason behind this could be that because of the presence of mordant there could be an effect on the fabric so that the pale yellow part of mahogany leaf extract might not work with its own flavor and the effect of mordant could be there. So, the combination of mordant and the mahogany leaf extract has some mixed effect but in the case of mordant less samples the case is different because of the absence of mordant. Of course, the red green scale has less effect by the raise of temperature because the mahogany leaf extract has less effect on the red color.

### 3.3 Uniformity Assessment

**Fig. 6:**
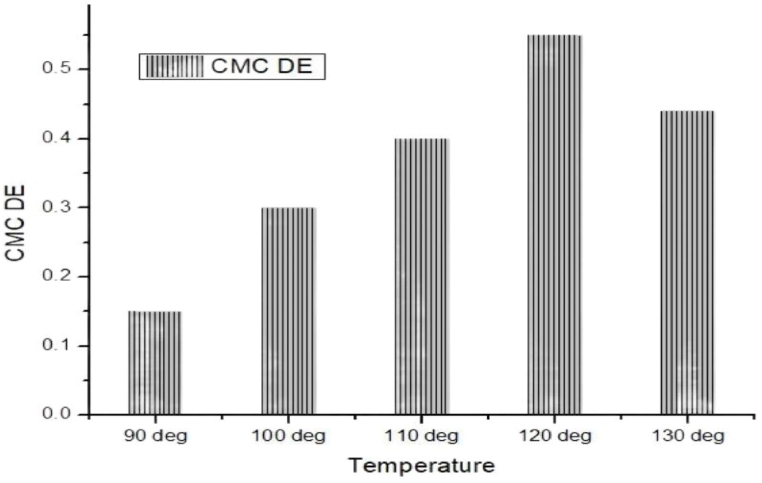
CMC DE color differences average value of mahogany leaf extract dyed sample without using mordant.

**Fig. 7:**
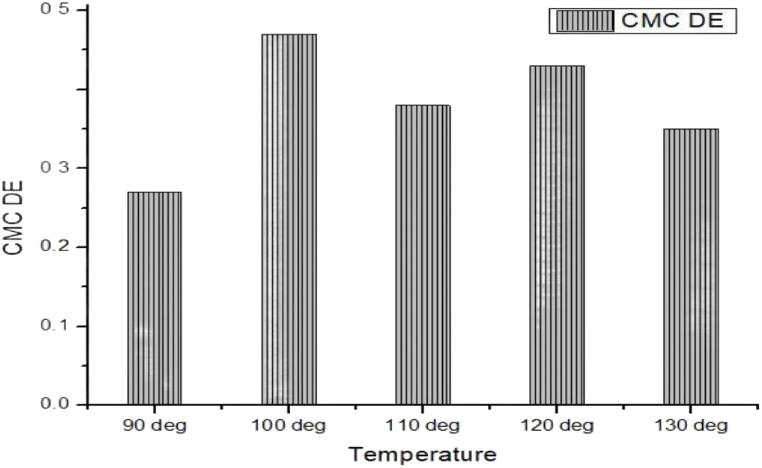
CMC DE color differences average value of mahogany leaf extract dyed sample by using mordant.

Table 3, Table 4 and Fig. 6, Fig. 7 are representing the evenness by CMC DE color differences. It is observed as per temperature at 90 °C the color difference at the six positions were very close to each other which refers to even shade. Again at increased temperature of 100 °C more or less similar results are experienced. Of course some positions are not that much similar but still it works. During more increasing of temperature at 110 °C the initial position 1 and position 5 & 6 more or less similar but in between position 2, 3 & 4 has some deviation but all the results are within ranges. The same way 120 °C & 130 °C temperature as the same color difference values at random six positions which is a presentation/affirmation of even shade of course which some deviation at some positions. The even shade is accounted for the dye uptake by the cotton modal blend at molecular level. It is understood that the triterpenoid groups are supposed to be attached with cellulose of cotton and also the monomeric content of modal as it is a semi-synthetic fibre. The constitutional monomer of modal is cellulose. Hopefully, the dye uptake by cotton and modal the dye uptake pathway of cotton and modal are thought to be more or less same which results as the near/close to color differences and the even shade.

**Table 3:** Determination of evenness by CMC DE color differences value of mahagony leaf extract dyed sample without using mordant.

CMC DE	Temperature	Position (1–2)	Position (1–3)	Position (1–4)	Position (1–5)	Position (1–6)	Position (1–7)
Without mordant	90 °C	0.18	0.05	0.15	0.31	0.10	0.15
	100 °C	0.27	0.14	0.12	0.25	0.49	0.53
	110 °C	0.48	0.27	0.30	0.15	0.46	0.75
	120 °C	0.46	0.35	0.54	0.82	0.71	0.43
	130 °C	0.49	0.28	0.32	0.56	0.47	0.57

**Table 4:** Determination of evenness by CMC DE color differences value of mahagony leaf extract dyed sample by using mordant.

CMC DE	Temperature	Position (1–2)	Position (1–3)	Position (1–4)	Position (1–5)	Position (1–6)	Position (1–7)
With mordant	90 °C	0.14	0.11	0.39	0.42	0.40	0.19
	100 °C	0.71	0.40	0.40	0.82	0.21	0.30
	110 °C	0.43	0.47	0.53	0.12	0.42	0.34
	120 °C	0.41	0.27	0.89	0.23	0.52	0.26
	130 °C	0.27	0.58	0.12	0.23	0.39	0.55

On the other hand, during the mordant treated natural dyed blend, it is observed that at distinguish positions the color differences are all within the ranges but still it was some random deviation also specially at temperature 120 °C and 100 °C which is not similar for the case of the mordant less samples. This data is inspiring for the mordant less samples that at the same temperature the color differences of the six positions are not that much debated. Only some few exceptions but it is evidently seen with mordant treated dyed samples that more deviations are experienced. The reason behind this could be that the mordant has its own effect during the fibre bonding with the mordant and it could be an effect on overall shade formation which is not possible in the case of the mordant less samples.

### 3.4 FTIR analysis

**Fig. 8:**
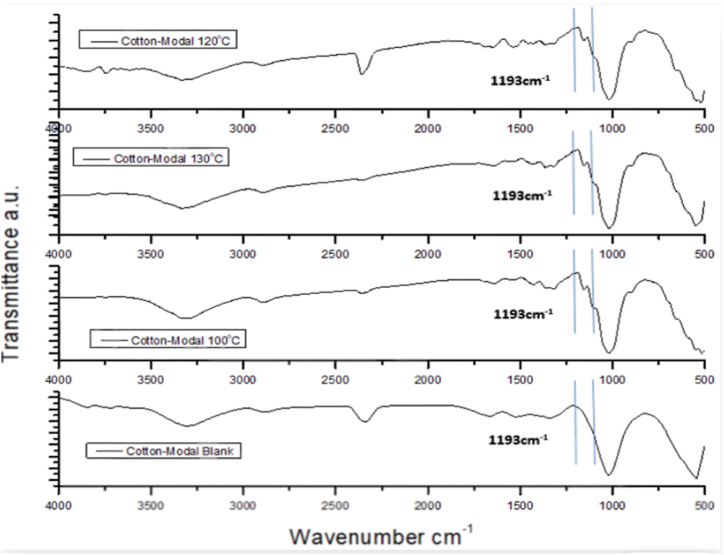
FTIR spectrum graph of cotton-modal blank and dyed cotton modal mahogany leaf extract dyed samples.

During dyeing with mahogany leaf extract dye, the sample exhibited the same characteristic peak at around 1200 cm^−1^ (1193 cm^−1^) as it is seen in Table 5 and Fig. 8, confirming the bonding of dye with fibre. It is known that the chemical components of mahogany leaf extract are mostly triterpenoids of limonoid class [10], where most triterpenoids are alcohol and can combine with sugars to form glycoside [31]. The repeating unit of cotton is cellubiose, where two β-glucose units are linked with 1–4 glucosidic linkage.

**Table 5:** FTIR report analysis of un-dyed cotton-modal blend, reference conventional dyed and mahogany dyed cotton-modal blends.

Characteristic band	Sample for FTIR analysis		
	Un-dyed cotton-modal blend	Reference conventional dyed	Mahogany dyed cotton-modal blend
H- bonded OH stretch	3537.45 cm^−1^ 3367.71 cm^−1^ 3327.21 cm^−1^	3495.01 cm^−1^ 3331.07 cm^−1^	3612.67 cm^−1^ 3342.64 cm^−1^
CH_2_ stretching	–	2875.86 cm^−1^	–
C=O stretching	–	–	–
H–*O*–H bending vibration of absorbed water molecules	1639.49 cm^−1^	1635.64 cm^−1^	1627.92 cm^−1^
CH_2_ scissoring	1427.32 cm^−1^	1425.40 cm^−1^	1425.40 cm^−1^
CH_2_ wagging	1317.38 cm^−1^	1313.52 cm^−1^	1315.45 cm^−1^
C–*O*–C stretching	–	1193 cm^−1^	1193 cm^−1^
C–O stretching	1026.13 cm^−1^	1026.13 cm^−1^	1026.13 cm^−1^
Amorphous cellulose	810.10 cm^−1^	867.97 cm^−1^	877.61 cm^−1^

### 3.5 Evaluation of color fastness

From Table 6, Table 7 it was experienced that the color fastness properties of mahogany leaves extract dyed cotton-modal blend experienced grading 4 and 4–5 on an average. For rubbing fastness, rating 4–5(dry) and 4 (wet) represented that the preponderance of dyes was fixed well on the fibres and surface residual dye was negligible. In terms of color fastness to wash, very good fastness was found due to buildup of covalent bond between dye-fibre interactions. The same way, color fastness to perspirations was exhibited very good fastness rating for alkali medium regarding color change and color staining. The constitutional component of mahogany leaf extract might have not that much stability to sustain under the radiation for long. However, the overall rating was promising.

**Table 6:** Color fastness properties of mahogany leaf extract dyed sample without using mordant.

	Temperature	Rubbing		Wash		Perspiration	
		Dry	Wet	Color Change	Color Staining	Color Change	Color Staining
Without Mordant	90 °C	4/5	4	4/5	4/5	4/5	4
	100 °C	4/5	4	4/5	4/5	4/5	4
	110 °C	4/5	3/4	4/5	4/5	4/5	4/5
	120 °C	4/5	3/4	4/5	4/5	4/5	4/5
	130 °C	4/5	3	4/5	4/5	4/5	4/5

**Table 7:** Color fastness properties of mahagony leaf extract dyed sample by using mordant.

	Temperature	Rubbing		Wash		Perspiration	
		Dry	Wet	Color Change	Color Staining	Color Change	Color Staining
With Mordant	90 °C	4/5	3	4/5	4/5	4/5	4
	100 °C	4/5	3/4	4/5	4/5	4/5	3/4
	110 °C	4/5	3/4	4/5	4/5	4/5	4
	120 °C	4/5	3	4/5	4/5	4/5	4
	130 °C	4/5	2/3	4/5	4/5	4/5	4

### 3.6 Bursting strength

From Table 8, Table 9, it is shown that bursting strength value of mahogany leave extract dyed cotton modal blend at temperature 90 °C, 100 °C, 110 °C, 120 °C & 130 °C with mordant are 351, 261, 302, 321 & 275 respectively. In the same way, the dyed blends without mordant reveal the bursting strength value of 363, 320, 339, 343 & 319 respectively. Whereas, the value of bursting strength of cotton modal blank fabric was 336. It is very clear that the strength was not deviated after dyed also it is known that cotton is not hampered below the temperature of 350 °C.

**Table 8:** Bursting Strength analysis of mahogany leaf extract dyed sample without using mordant.

	Temperature	Energy to Peak (N.m)	Dist. @ Peak (mm)	Bursting Strength (N)	Energy to Break (N.m)
	Cotton-Modal Blank	1.362	14.787	336.900	1.466
Without Mordant	90 °C	1.510	15.318	363.900	1.510
	100 °C	1.309	14.998	320.000	1.445
	110 °C	1.442	15.310	339.100	1.490
	120 °C	1.411	15.030	343.900	1.411
	130 °C	1.307	14.954	319.900	1.307

**Table 9:** Bursting Strength analysis of mahogany leaf extract dyed sample by using mordant.

	Temperature	Energy to Peak (N.m)	Dist. @ Peak (mm)	Bursting Strength (N)	Energy to Break (N.m)
	Cotton-Modal Blank	1.362	14.787	336.900	1.466
With Mordant	90 °C	1.447	15.030	351.000	1.447
	100 °C	1.023	14.084	261.010	1.023
	110 °C	1.228	14.623	302.630	1.321
	120 °C	1.297	14.645	321.100	1.297
	130 °C	1.133	14.448	275.970	1.133

## 4 Conclusion

Using sources for raw materials from nature is more desirable and acceptable to science community. Process minimization is another aspired phenomenon for researchers to achieve an energy saving cost friendly method. The proposed method of implementation of mahogany dyes for 50 % cotton and 50 % modal blended knit fabric dyeing in this study was fulfilled the above criteria. In this research work, covalent bond was found between cellulose and dye molecule that can be confirmed at FT-IR analysis. The sample exhibited the same characteristic peak at around 1200 cm^−1^, confirming the bonding of dye with fibre. As mahogany leaf extract consists of suitable functional groups, the dyed samples showed perfect fastness properties for wash, rubbing and perspiration as well. K/S value was found 1.65 in case of mordantless dyed sample that indicated higher depth of shade even in the absence of mordant. This accounted for the huge alcoholic content of limonoid group (triterpenoid) that is able to form glycoside linkage with glucose. Evenness of shade was affirmed by receiving closer data of CMC DE and k/s values from random check point within the same sample. Finally it can be concluded that using swietenia mahogany as natural dye for the coloration of blend fabric is very promising and have a great future and potentiality in the world of textile coloration in sense of eco-friendliness as well as energy and cost saving coloration of textiles.

## Limitation

This work was done for only 10 % mahogany dye recipe and at a fixed time and different temperature respectively 90 °C, 100 °C, 110 °C, 120 °C & 130 °C in all cases. Only cotton-modal blended knit fabric was used, scope for other natural and synthetic single or other blend fibers knit and woven fabrics also could be used. Light fastness testing wasn't performed because of not working condition of the machine. Only lab/sample dyeing was done, bulk production was not executed.

## Data availability statement

The authors confirm that the data and materials supporting the findings of this research are available within the article.

## CRediT authorship contribution statement

**Md Abdul Hannan:** Writing – original draft, Supervision, Resources, Investigation, Data curation, Conceptualization. **Md Faridul Islam:** Methodology, Investigation, Formal analysis, Data curation. **Mohammad Bellal Hoque:** Writing – review & editing, Visualization, Methodology, Formal analysis.

## Declaration of competing interest

The authors declare that they have no known competing financial interests or personal relationships that could have appeared to influence the work reported in this paper.
